# Development of an inactivated, oral immunogenic product against post-weaning colibacillosis caused by enterotoxigenic *Escherichia coli* in piglets

**DOI:** 10.1186/s12917-026-05846-5

**Published:** 2026-09-02

**Authors:** Alessia Inglesi, Joel Filipe, Giulia Valli, Federica Riva, Paola Dall’Ara, Gianfilippo Alessio Clemente, Mario D’Incau, Ivonne Laura Archetti, Valentina Lorenzi, Francesca Battioni, Sara Rota Nodari, Matteo Tonni, Antonio Marco Maisano, Flavia Guarneri, Massimo Amadori, Francesca Fusi

**Affiliations:** 1 Department of Veterinary Medicine and Animal Sciences, University of Milan, Lodi, 26900 Italy; 2 Istituto Zooprofilattico Sperimentale della Lombardia e dell’Emilia-Romagna (IZSLER), Via A. Bianchi, 9, Brescia, 25124 Italy; 3 ATS Brescia, Viale Duca degli Abruzzi, 15, Brescia, 25124 Italy; 4 Italian Network of Veterinary Immunology, Brescia, Italy

**Keywords:** Enterotoxigenic *Escherichia coli* (ETEC), Fimbrial antigens (F4/F18), Post-weaning diarrhea (PWD), Immune response, Piglets

## Abstract

**Background:**

Post-weaning diarrhea (PWD) caused by enterotoxigenic *Escherichia coli* (ETEC) expressing F4 and F18 fimbriae remains a major challenge in pig production, contributing to economic losses and increased antimicrobial use. This study aimed to preliminary evaluate the immunogenic potential of an orally administered formulation comprising heat-inactivated ETEC strains expressing F4 and F18 fimbriae, combined with low-dose recombinant human interferon-alpha (IFN-α) as a mucosal adjuvant, in piglets. Piglets from two different litters were allocated into two experimental groups: a treated group (T) receiving the inactivated ETEC formulation with IFN-α for 26 days, and a control group (C) receiving only IFN-α. According to the farmer, the sows had not been vaccinated. Immune responses were evaluated in the sows colostrum and piglet serum, saliva, and feces by ELISA. In mesenteric lymph nodes anti-F4/F18 IgA and IgG antibodies were quantified by ELISPOT.

**Results:**

The inactivated product preserved fimbrial antigenicity and remained sterile. Colostrum from both sows displayed elevated levels of fimbriae-specific IgA and IgG despite neither sow being vaccinated against *E. coli.* Treated piglets showed a transient serum IgA increase against F4 whereas serum IgG levels were often comparable to the controls. Notably, elevated mucosal IgA responses were observed in saliva and feces against both F4 and F18 (*P* < 0.01) in T group, accompanied by enhanced IgA-secreting B-cell activity in mesenteric lymph nodes. ELISA validation confirmed high assay reproducibility (R² > 0.98; CV < 10%). ELISPOT analysis underscored the adjuvant role of F4 and IFN-α in stimulating mucosal immunity.

**Conclusions:**

Our preliminary findings may represent a promising strategy to control PWD and reduce antimicrobial use in pig production.

**Supplementary Information:**

The online version contains supplementary material available at https://doi.org/10.1186/s12917-026-05846-5.

## Introduction

Enteric colibacillosis is one of the most severe diseases threatening both neonatal and weaned piglets, implying important economic losses as a result of morbidity, mortality, reduced growth rate and cost of therapies [1–3]. Mortality rates vary depending on *E. coli* pathogenicity and piglet age. During the critical period of weaning the piglet gut undergoes physiological, nutritional, immunological, and inflammatory modifications leading to a microenvironment that promotes *E. coli* proliferation [3, 4]. This may lead to porcine post-weaning diarrhea (PWD), a multifactorial condition that typically develops within the first two weeks after weaning, with clinical signs most commonly observed during the first week (approximately 3–10 days post-weaning) [5–7]. In this disease the presence of ETEC is necessary but not sufficient to induce clinical signs. PWD typically results in average mortality rates of 9.4–12.6%, but acute outbreaks have been reported to cause losses up to 20–30% over a 1- to 2-month period [8]. PWD is mainly caused by the proliferation of ETEC strains [9]. ETECs represent about 65 to 70% of diarrhea-causing bacterial agents and are characterized by the expression of long fimbriae, that bind to specific receptors present on the brush border of enterocytes in the small bowel. ETECs primarily colonize the jejunal and ileal regions, facilitating pathogen persistence and toxin-mediated fluid hypersecretion [10–12]. Five fimbriae adhesins, F4 (K88), F5 (K99), F6 (987P), F41 and F18 (F107) have been described as colonization factors in porcine ETEC strains [8, 10, 13–16]. F4, responsible for both neonatal disease and PWD, presents three serological variants (F4ab, F4ac or F4ad), F4ac being the most widespread in Europe [17–20].On the other hand, F18 fimbriae exists in two serotypes: F18ab and F18ac, usually associated with edema disease and PWD, respectively [10, 14]. After the colonization of the gut epithelial barrier, these bacteria produce enterotoxins, including the thermolabile toxin (LTI) and two heat-stable toxins (STa and STb), that induce electrolytes and fluid release into the lumen leading to severe secretory diarrhea [14, 21]. The use of antibiotics is a commonly used method of treating porcine colibacillosis during the post weaning period [21]. This massive use of antibiotics in pig husbandry represents a broader problem that also affects human health, because of the spread of antimicrobial-resistant (AMR) *E. coli* and other microorganisms [22, 23]. Considering the serious impacts on both human and animal health, vaccine-based control strategies are badly needed. To this purpose, oral immunizations are most suited due to reduced invasiveness and handiness; also, they are more likely to boost the mucosal immune response, that results more effectively in the protection against such an infection [24, 25]. Oral vaccines aim to stimulate and subsequently activate mucosal immunity via the gut-associated lymphoid tissue (GALT), resulting in the local production of secretory IgA (SIgA) antibodies to *E. coli* adhesins; such antibodies prevent pathogenic F4 + and F18 + ETECs from attaching to their receptors, thereby reducing the occurrence of clinical signs of PWD [1, 26, 27]. This preliminary study aimed to develop a novel, oral, safe, inactivated immunogenic product based on selected ETEC strains, and to assess its immunogenicity in piglets around weaning.

## Materials and methods

### Preparation of the immunizing product

Two *E. coli* strains were selected from the internal biobank of the Istituto Zooprofilattico Sperimentale della Lombardia e dell’Emilia-Romagna “Bruno Ubertini” (IZSLER, Brescia, Italy) for the development of the immunogenic product: one expressing somatic antigen O149 (genome: F4+, LTI + and STb+, internal code 2019/325282/3) and another one expressing somatic antigen O139 (genome: F18+, internal code 2019/333386/1) [2, 21, 28].The two strains were stored in 20% glycerol at -80 °C. The production of the immunogenic product was carried out at the Vaccines and Reagents Laboratory in IZSLER. From each strain, a starter culture was developed and was inoculated into sucrose-enriched Brain Heart Infusion (BHI) broth. Cultures were placed in incubator at 37 °C with moderate shaking for 24 h. At the end of a 24-hour incubation, the culture broth was sampled for a purity check using Gram staining under a microscope, as well as a dedicated PCR (see next chapter) and a hot slow agglutination test for O-antigen detection as previously described [29, 30] on *E. coli* F4/F18 fimbrial antigen, purchased from Ghent University (Ghent University, Ghent, Belgium).

Once purity was confirmed, bacterial concentration was assessed using the WPA CO8000 Biowave cell density meter (Biochrom Ltd., Cambridge, England) inside a laminar flow hood. This procedure was necessary to determine the original concentration of bacterial cells; these were further concentrated to achieve a final bacterial concentration of 10^10^ CFU/mL (following Porter, 1973 [31]).

Therefore, the bacterial culture was centrifuged twice at 3,000 x g for 45 min. The resulting pellet of each strain was resuspended in sterile distilled water to achieve a final concentration of 10^10^ CFU/mL (as described in Porter, 1973 [31]) and heat-inactivated in a water bath at 70 °C for 60 min. The inactivated suspension was divided into aliquots and stored at -20 °C pending in vivo experimentation. One aliquot was stored at -80 °C for enzyme-linked immunosorbent assay (ELISA) to verify the integrity of the F4 and F18 adhesins. The inactivated product was eventually supplemented with human interferon alpha (IFN-α) purchased in lyophilized form from the National Institute for Biological Standards and Control (NIBSC; https://www.nibsc.org/) as immune response adjuvant. Each lyophilized ampoule of IFN-α contained 38,000 International Units (IU). According to the manufacturer’s instructions, the IFN-α was reconstituted by adding 1 mL of sterile distilled water, obtaining a stock solution with a concentration of 38,000 IU/mL. The stock solution was divided into several aliquots, each of which was diluted with distilled water to obtain a working concentration of 100 IU/mL. During the experimental trial on piglets, 1 mL of this preparation was administered daily to each litter (both treated and control), mixed with the inactivated product and with powdered milk in drinking water, in order to ensure an actual intake of 1 IU/kg body weight/day, based on the average fluid consumption of piglets. The final immunizing product administered to the piglets consisted in a mix of F4 + inactivated *E. coli*, F18 + inactivated *E. coli* and IFN-α.

#### Multiplex PCR for the detection and confirmation of ETEC F4 and F18 genes in bacterial culture

To confirm strain identity, genomic DNA was isolated from bacterial broth cultures according to the heat lysis protocol of Casey and Bosworth [32] with modifications adapted for this study. One frozen aliquot from each bacterial strain culture was thawed and then centrifuged at 12,000 × g for 5 min and washed with 1 mL of PBS. The resulting pellet was resuspended in 500 µL of 6% Chelex-100 (Bio-Rad Laboratories, Hercules, CA, USA), and the suspension was incubated at 56 °C for 20 min, vortexing every 5 min. Subsequently, samples were incubated at 97 °C for 10 min, immediately cooled on ice for 2 min, and centrifuged again at 12,000 × g for 5 min. The resulting supernatant was subjected to multiplex PCR for the screening of virulence factors, including those encoding for the toxins LTI⁺, STaP, STb, Stx2e and the adhesins F4 (K88), F5 (K99), F6 (987P), F41, F18, following the protocol described by Casey and Bosworth [32]. Briefly, the PCR reaction mix contained 9 primer pairs at a concentration of 0.5 µmol each, 0.2 mmol of deoxyribonucleotide triphosphate mix, 1× reaction buffer, 5 mmol MgCl_2_, and 2.5 units of Taq polymerase, in a final volume of 20 µL. PCR amplification was performed using a SureCycler 8800 Thermal Cycler (Agilent Technologies, CA, USA) under the following thermal conditions: an initial denaturation at 94 °C for 10 min; 30 cycles of denaturation at 94 °C for 30 s, annealing at 55 °C for 45 s, and extension at 72 °C for 90 s, with the extension time increased by 3 s at each cycle; followed by a final extension at 72 °C for 10 min. The amplicons were analyzed by capillary electrophoresis using the QIAxcel system (Qiagen, Hilden, Germany).

#### Sterility test

A sterility test was conducted to detect any bacterial contamination in the heat-inactivated bacterial suspensions. Briefly, 1 mL of the immunizing product was inoculated into test tubes containing BHI Broth, Sabouraud Broth, Trypticase Soy Broth (Biolife Italiana, Milan, Italy), and Thioglycolate Broth (Microbiol s.r.l. Diagnostici, Catania, Italy), respectively, and then incubated at 37 °C. After 72 h, the sterility of the samples was assessed based on the absence of turbidity in the media, indicating no microbial growth and confirming the effectiveness of the heat inactivation process.

#### Fimbriae integrity assay

An indirect ELISA assay was developed to test for the presence and structural integrity of F4 and F18 fimbriae in the inactivated product. Aliquots of the immunizing product stored at -80 °C were thawed at 4 °C and centrifuged at 10,621 × g for 10 min at 4 °C. The pellet was discarded and the supernatant was concentrated at different levels (1X, 5X, 10X, and 20X) by performing three consecutive centrifugation cycles at 1,700 × g for 10 min each, using Amicon^®^ Ultra 50 kDa MWCO filtration devices (Millipore Corp., MA, USA). For the ELISA, 96-well MaxiSorp plates (Nunc^®^ MaxiSorp MicroWell™, Merck Millipore, Burlington, MA, USA) were directly coated with 50 µL/well of fimbriae-containing supernatants at each concentration level, alongside negative controls (PBS only) and positive controls (purified F4 or F18 fimbriae at 25 µg/mL in PBS). Plates were incubated for 2 h at 37 °C to allow the fimbriae to adsorb onto the well surface. After incubation, the coating solution was removed, and wells were incubated with PBS containing 0.2% Tween 80 for 1 h at 37 °C to reduce nonspecific binding [33, 34]. Subsequently, 50 µL/well of anti-fimbriae monoclonal antibody (Monoclonal antibody *E. coli* F18 and Monoclonal antibody *E. coli* F4 purchased from Ghent University, Ghent, Belgium) at 1 µg/mL in PBS supplemented with 0.2% Tween 20 and 3% bovine serum albumin (BSA) was added to half of the replicates, while the other half received only PBS with 0.2% Tween 20 and 3% BSA as negative antibody controls. These mAbs recognize the conformational epitopes of F4 and F18. After a 1-hour incubation at 37 °C, plates were washed three times with 300 µL/well of 0.05% PBS-Tween 20 (PBS-T). Next, 50 µL/well of horse radish peroxidase (HRP)-conjugated anti-IgG antibody (IDEXX PRRS X3 Ab / PRRS 2XR ELISA, IDEXX Laboratories, Westbrook, ME, USA) diluted in PBS with 0.2% Tween 20 and 3% BSA was added, and the plates were incubated for another hour at 37 °C. After three additional washes with PBS-T, 50 µL of TMB substrate solution (3,3′,5,5′-tetramethylbenzidine; IDEXX Laboratories, Westbrook, ME, USA) was added to each well. The plate was incubated for 15 min at room temperature in the dark, and the reaction was then stopped by adding 50 µL of IDEXX stop solution to each well (IDEXX Laboratories, Westbrook, ME, USA). Absorbance was measured at 650 nm using a Sunrise™ spectrophotometer (Tecan, Switzerland). A reaction was considered positive when the OD value was greater than the mean OD of the negative control plus three standard deviations.

### Oral administration of the immunogenic product to piglets

The experimental trial was performed at the IZSLER animal facility, after approval of the project by the Italian Ministry of Health (no. 1230/2020-PR, prot. 0028530-31/12/2020-DGSAF-MDS-P), in compliance with the current legislation on the protection of animals used for scientific purposes (Italian Legislative Decree no. 26 of March 2014, transposition of the European Directive 2010/63/EU). Two Large White x Landrace sows, sharing the same expected farrowing date and from the same fattening pig farm located in Brescia province, Italy, were selected for the trial of the immunizing product. The farm of origin was selected from those free of clinical outbreaks referred to ETEC *E. coli* strains, as confirmed by diagnostic laboratory controls and by declarations of the farm’s veterinary practitioners. The circulation of the pathogen in the farm cannot be excluded, since it is a ubiquitarian microorganism. The sows were raised under the same environmental and management conditions; moreover, they were housed in the same group. The sows were not vaccinated against any *E. coli* antigen to avoid any possible interference with the test results. Before starting the trial, fecal samples were collected from both sows and tested by Multiplex PCR on a SureCycler 8800 Thermal Cycler to ensure they were not carriers of ETEC strains expressing F4 and F18 fimbriae, following the protocol previously described by Casey and Bosworth [32]. Samples were taken at weekly intervals. On day 70 of the presumed pregnancy, the sows were transferred to the IZSLER animal facility and housed in the same pen until one week before farrowing. During this period, they were fed *ad libitum* the same feed used in the herd of origin. One week before the expected farrowing date, the sows were separated into two isolation rooms, with the same environment. The litter of one sow (13 piglets) was selected for the administration of the immunizing product as treated group (group T); while the litter of the other sow (15 piglets) was selected as the control group (group C). No litter balancing was performed to avoid contamination. Both groups of piglets were fed a milk replacer (Denkapig, Denkavit) until reaching 32 days, when a complete first weaning phase diet was gradually introduced (Suistar 10, Veronesi). That day the piglets were separated from the sows but remained in their rooms until day 54. They were then moved into two separate outdoor pens, where they stayed until the end of the study. Piglets in group T received the following treatment: 1 mL of IFN-α (100 IU/mL) combined with 3 mL of product per liter of drinking water. The product consisted of 1.5 mL of inactivated F4 bacterial antigen and 1.5 mL of inactivated F18 bacterial antigen. Administration started on day 14 of life and continued for 26 consecutive days.

To improve palatability, powdered milk (50 g/L) was initially added to the solution, and its concentration was gradually reduced each week until it was completely replaced with water. As the volume of drinking water intake increased, the amount of product preparation was proportionally adjusted, maintaining a constant ratio of 3 mL/L; the IFN-α dose was kept fixed at 1 mL (100 IU/mL) per day. The Group C was treated in the same manner as Group T, receiving drinking water supplemented with powdered milk, but without the product preparation. Therefore, only with 1 mL of IFN-α (100 IU/mL) per day for the entire duration of the trial period. In addition, during the experimental study, we did not observe significant differences between treated and control piglets in terms of body weight or average daily weight gain at any sampling time. At the end of the trial between 34 and 42 days after the last administration of the immunizing product, all piglets were sacrificed for necropsy and sample collection. Gross examination of the whole carcass revealed no alterations attributable to infections or other pathological conditions.

### Samples collection and preparation

Various biological samples were collected from sows and piglets at specific time points throughout the study. At T0 (within the first postpartum hours), colostrum was collected from both sows. The following samples were collected from all the piglets: individual saliva and fecal samples at T1 (14 days of age and vaccination onset); individual saliva, feces, and blood samples at T2 (28 days of age and 14 days post-treatment), individual saliva and fecal samples at T3 (47 days of age and 33 days post-treatment); individual saliva, feces, blood and mesenteric lymph nodes at T4 (73–85 days of age and 60 days post-treatment) (the time of culling).

Colostrum samples were centrifuged two times (first: 5,204 × g for 15 min, and second: 20,800 × g for 60 min, both at 4 °C) to eliminate fat and cells. Acellular defatted colostrum was transferred to clean tubes after each step, and then immediately frozen at − 80 °C.

Blood samples (~ 5 mL per piglet) were collected from the external jugular vein into Vacutest^®^ tubes without anticoagulant (Kima s.r.l., Arzergrande, Italy) and centrifuged (1,700 × g for 15 min) to obtain serum. Serum was stored at − 20 °C.

Individual saliva samples were collected from piglets using cotton swabs (Salivette^®^ system, Sarstedt AG & Co. KG, Nümbrecht, Germany), recovered after 2 min, and centrifuged (3,234 × g for 10 min at room temperature (RT); the resulting saliva was stored at − 20 °C until analysis. Additionally, pooled saliva samples were daily collected over 21 days (from the fourth day of treatment) during and after treatment using frayed hemp ropes hung in pens for up to 3 h/day, manually squeezed to extract absorbed saliva in tubes. The saliva tubes were centrifuged (10,400 × g for 10 min) to eliminate the cells and stored at − 20 °C until further use.

Fecal samples were collected from piglets via rectal stimulation using swabs soaked in Luan^®^ 2.5% (Molteni & C, Scandicci, Italy) and stored at -20 °C in sterile tubes. The preparation of fecal samples for ELISA assay followed the method described by Wang et al. (2014) [35] using a 1:4 ratio (1 g of feces in 3 mL of PBS with 0.05% Tween 20). Briefly, the suspension obtained was centrifuged immediately at 6,000 × g for 25 min. The supernatant was then collected and stored at − 20 °C until the day before use. On the day prior to testing, fecal samples were thawed in at 4 °C overnight. The following morning, the samples were centrifuged again at 4,000 × g for 10 min. The resulting supernatants were used directly in the ELISA assays.

Mesenteric lymph nodes were collected from euthanized piglets to isolate mononuclear cells through the modified protocol described by Razzuoli et al. [36]. Briefly, lymph nodes were collected and immediately stored into a 50 mL sterile tube containing RPMI-1640 (Gibco™, Thermo Fisher Scientific, Waltham, MA, USA) supplemented with a 5X antibiotic mixture: penicillin (250 µg/mL), streptomycin (250 µg/mL) and neomycin (50 µg/mL) and transported within 2 hours to the laboratory at 4 °C. The lymph nodes were washed in sterile RPMI-1640, placed in a sterile Petri dish to clean them from the excess of connective tissue and fat. Lymph nodes were smashed through a nylon 70 μm cell-strainer with RPMI-1640 supplemented with 10% fetal bovine serum (FBS) and 1% Penicillin (100 IU/mL) - Streptomycin (100 µg/mL) in a final volume of 50 mL. Mononuclear cells were separated by centrifugation on a layer of Histopaque^®^ 1.077 (Sigma-Aldrich, St. Louis, MO, USA) at 1,100 x g for 25 min at 20 °C. The mononuclear ring was collected and washed in 50 mL PBS (Ca^2^+ and Mg^2^+ free) 1X and centrifuged at 330 x g for 10 min at 20 °C. Before assessing cell concentration, the cell pellet was re-suspended in 10 mL of RPMI-1640 medium (supplemented with 10% FBS and 1X antibiotic mixture). The cells were centrifuged and divide in aliquots at 6 × 10^6^ viable cells/mL in a freezing medium (50% RPMI-1640, 40% FBS and 10% DMSO) and stored at − 80 °C until an ELISPOT assay.

### Development and Optimization of the Sandwich ELISA

A criss-cross serial dilution test was performed to optimize reagent concentrations for a home-made sandwich ELISA targeting anti-F4 or F18 antibodies. Capture monoclonal antibody (mAb) anti-F4 or F18 for coating was used at 1 µg/mL based on literature recommendations [37], which suggested an optimal range of 0.5–8 µg/mL. This approach allowed us to compare different assay conditions and identify the most suitable concentrations (purified fimbriae at 5 µg/mL; anti-pig IgA and IgG conjugates 20 ng/mL; Streptavidin 20 ng/mL). As for samples, colostrum was diluted 1:50 and sera 1:20; pooled and individual saliva and feces were employed undiluted.

### Sandwich ELISA assay for F4/F18-specific IgG and IgA

Porcine anti-F4/F18 IgA and IgG were measured by a sandwich ELISA assay using the ELISA Starter kit (Bethyl™ Laboratories, Montgomery, TX, USA). Monoclonal antibodies and fimbriae were purchased from Ghent University (Merelbeke, Belgium). 100 µL of properly diluted monoclonal antibodies IMM01 for F4 [33] and IMM02 for F18 [38], respectively, were used for the coating on high-binding microtiter plates (Nunc MaxiSorp™, Serving Life Science, Denmark) at 1 µg/mL. Plates were incubated overnight at RT. After the coating step, plates were washed three times with phosphate-buffered saline containing 0.05% Tween-20 (PBS-T). Plates were blocked with 5% BSA in PBS-T (200 µL per well) for one hour at RT to minimize non-specific binding. Subsequently, plates were washed three more times with PBS-T. Then, 100 µL/well of F4 or F18 purified fimbriae at 5 µg/mL were added, and plates were incubated at RT for one hour. The plates were washed four times with PBS-T. Afterwards, different dilutions for each matrix (colostrum, serum, feces, individual saliva, pooled saliva) were tested. A volume of 100 µL of each sample was added to the antigen-coated wells, and the plates were incubated for one hour at 37 °C with gentle shaking. After incubation, plates were washed five times with PBS-T to remove unbound material. Plates were reacted with 100 µL of secondary antibody (Goat anti-pig IgG-biotinylated or Goat anti-pig IgA-biotinylated; 1:50.000, Bethyl™ Laboratories, Montgomery, TX, USA) for one hour at RT to detect IgG or IgA against *E. coli* fimbriae. Subsequently, the ELISA plates were washed four times with PBS-T; the secondary antibody was reacted with Streptavidin (peroxidase/HRP conjugated, 1:25.000) (Elabscience^®^, Houston, TX, USA) and the plates were further incubated for 30 min at RT. Color development was achieved by adding 100 µL of 3,3′,5,5′-tetramethylbenzidine (TMB) substrate solution to each well. The reaction was allowed to proceed for 15 min at RT in the dark and was stopped by adding 50 µL/well of 0.18 M sulfuric acid. Optical density (OD) was measured at 450 nm using a microplate reader (MultiSkan FC, Thermo Fisher, Waltham, MA, USA). Negative controls (PBS-T without sample) and internal positive controls (colostrum samples) were included on each plate to check the accuracy of the assay. All samples were tested in duplicate; inter-assay and intra-assay variability were minimal, with coefficients of variation (CV) consistently below 10%. Since this in-house ELISA was developed as a qualitative assay to compare antigen-specific antibody levels between experimental groups, no diagnostic cut-off value was established to classify samples as positive or negative.

### ELISPOT assay for evaluating IgA antibody-secreting B cells

IgA-secreting cells in the mesenteric lymph nodes of piglets were analyzed using the Pig IgA Single-Color ELISPOT assay (Immunospot^®^ Cellular Technology Limited, Shaker Heights, OH, USA). The following procedure was employed: the lymph node cells were rapidly thawed at 37 °C in a water bath, immediately resuspended in 5 mL of fresh RPMI media with 10% FBS and 1:100 of antibiotics (Penicillin (100IU/mL) - Streptomycin (100 µg/mL) and centrifuged at 400 x g for 7 min. They were then resuspended again in 1 mL of RPMI medium, and the viability was determined by Trypan blue staining. At least, 8 × 10^5^ cells/well from each animal were resuspended in complete growth medium with 10% FBS and reacted with medium (no stimulation); polyclonal stimulus (Human B-Poly-S™ 1000X, Immunospot^®^ Cellular Technology Limited, Shaker Heights, OH, USA), F4 (2 µg/mL) and F18 (2 µg/mL) for 5 days at 37 °C in 5% CO_2_ in 96-well plates. The day before the ELISPOT assay, PVDF plates were activated by adding 15 µL/well of 70% EtOH and immediately washed twice with 180 µL PBS according to the manufacturer’s directions. PVDF plates were coated overnight at 4 °C with 80 µL porcine IgA capture antibody solution and finally wrapped in parafilm. The day after, the capture antibody solution was discarded, and plates were washed with sterile PBS. To block the residual reactive sites, plates were incubated at RT for one hour with complete assay medium (RPMI 1640 with 10% FBS, 1% L-glutamine, 1% Penicillin-Streptomycin, and 2-mercaptoethanol 50 µM). While the plates were incubated, cell suspensions were centrifuged at 400 x g for 7 min at 4 °C, and the cell pellet was resuspended in assay medium and plated at a final concentration of 4 × 10^5^ cells/well (100 µL) in duplicate on previously coated PVDF plates for four hours at 37 °C with 5% CO_2_. After incubation, plates were washed twice with PBS, then twice with PBS-T. Each time, the content was carefully removed avoiding to dry out the membrane. Plates were incubated for 2 h in the dark at RT with biotin-labelled anti-pig IgA detection antibody solution. After two washes with PBS-T, plates were incubated for 30 min in the dark at RT with Streptavidin-Alkaline Phosphatase solution (SA-AP 1:1000). Finally, plates were washed twice with distilled water and stained with blue substrate solution (CTL-TrueBlue) for 10 min in the dark at RT. After rewashing with tap water, plates were dried overnight in the dark, and spots were identified and counted by a CTL-Immunospot S6 Universal Analyzer (Immunospot^®^ v.7.0.22.1, Cellular Technology Limited, Shaker Heights, OH, USA).

### Statistical analyses

Statistical analysis was performed using Graph Pad Prism 9.3.1 (La Jolla, CA, USA). The treated and control groups were analyzed according to sampling time (T0-T1-T2-T3-T4). Data were first analyzed by descriptive statistics and then statistical tests were applied. The descriptive analysis included both individual test results and results in the form of mean ± 1 standard deviation (SD). Normal or non-normal distribution of the data was assessed using the Shapiro-Wilk test, and parametric or non-parametric tests were applied. Accordingly, to evaluate statistically significant differences in more than two groups of results at different sampling times, the Kruskal-Wallis test or the ANOVA test was applied. Statistical analysis on two groups was performed using either the Student’s *t* or the Mann-Whitney test; the Chi-square test was employed to investigate the prevalence of responses in the ELISPOT assay. Antibody assay results are presented as Box and Whisker plots, showing median, interquartile range, and minimum/maximum values [39]. The results were considered statistically significant with a p-value < 0.05. In the ELISPOT assay, negative values (subtracting the mean of the unstimulated wells from that of the antigen-stimulated wells) were converted to zero, for data analysis as they were interpreted as null responses.

## Results

### Results of the quality tests on the immunizing product

The application of Gram staining and hot slow agglutination tests provided unequivocal confirmation that the broth cultures consisted of pure *E. coli*. The sterility test was also successful as there was no growth of contaminating microorganisms in the heat-inactivated product. To confirm the integrity of the fimbriae, a highly specific ELISA test was performed using mAbs that recognize the conformational epitopes of F4 and F18. The reaction was considered positive when the OD value was greater than the mean OD of the negative control plus three standard deviation (Table S1).

#### Colostrum IgA and IgG

Two colostrum samples from both sows were tested, revealing consistently elevated IgG and IgA levels against F4 and F18 antigens (Table S2). Therefore, the colostrum from the sow of treated piglets was selected as the positive control due to its high and consistent positive values. This sample was diluted 1:50 and used in duplicate wells for each ELISA on all kinds of matrices.

#### Systemic response to oral immunization

**Fig. 1:**
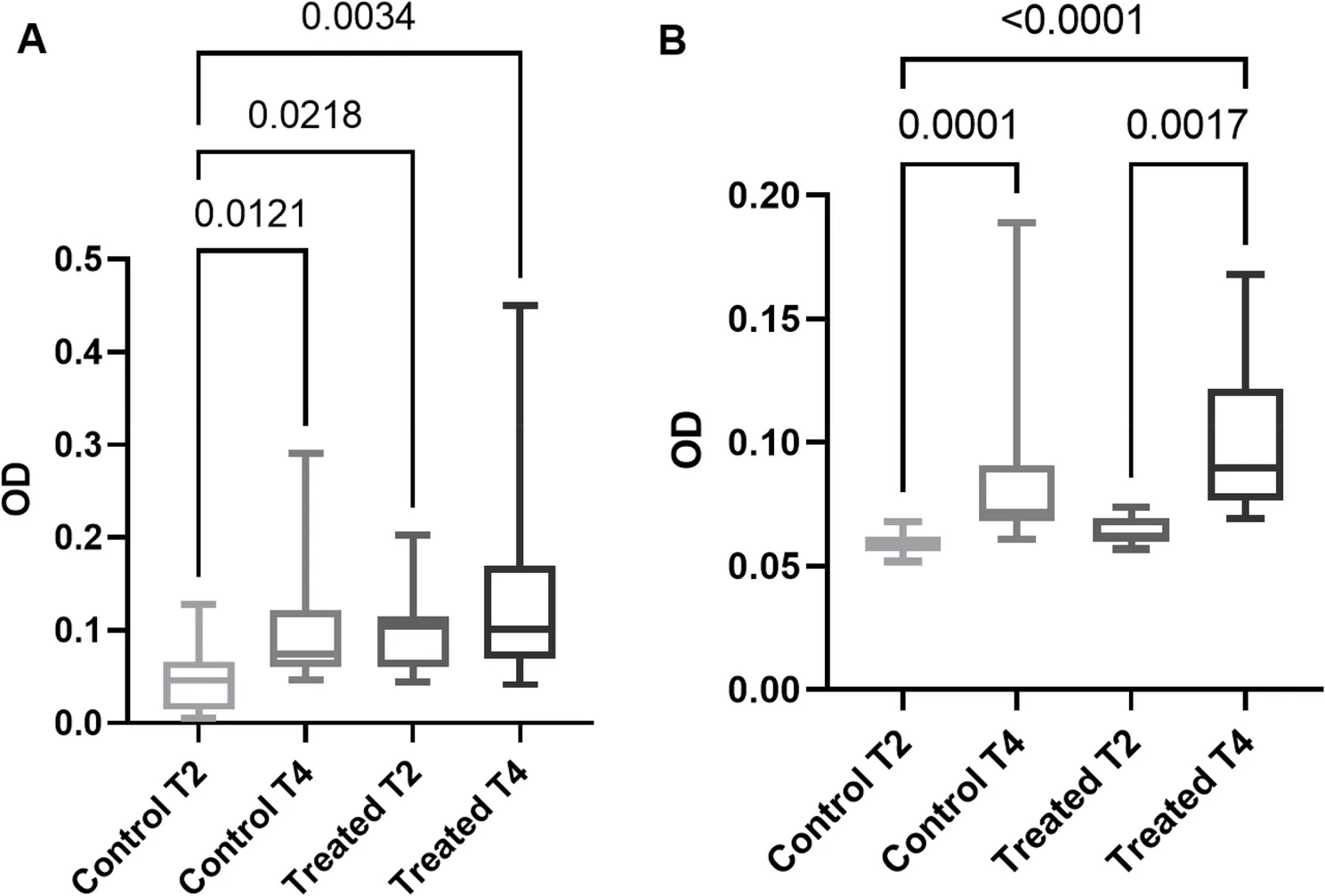
(**A**) IgA serum levels in treated and control piglets for F4; (**B**) IgA serum levels in treated and control piglets for F18

Serum samples were collected at T2 and T4 to evaluate fimbriae-specific IgA and IgG. Our results showed serum IgA absorbance values close to the assay background throughout the study period. There was evidence of a serum IgA response of treated piglets to F4 but not for F18 at T2, two weeks after the beginning of the treatment (Fig. 1A). Time-dependent variations in serum IgA levels of treated group were observed for both F4 and F18 at both time points (T2 and T4); however, similar or greater changes were also detected in the control group (Fig. 1A, B*).* The difference between control and treated groups is lost at T4 (Fig. 1A).

**Fig. 2:**
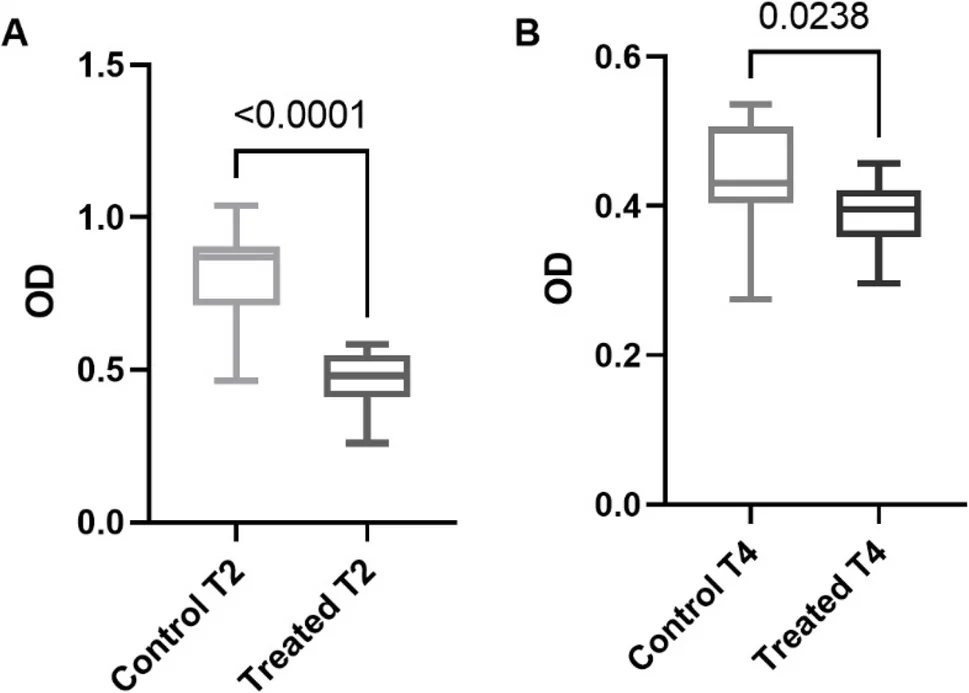
(**A**) IgG serum levels in treated and control piglets for F4 at T2; (**B**) IgG serum levels in treated and control piglets for F4 at T4

**Fig. 3:**
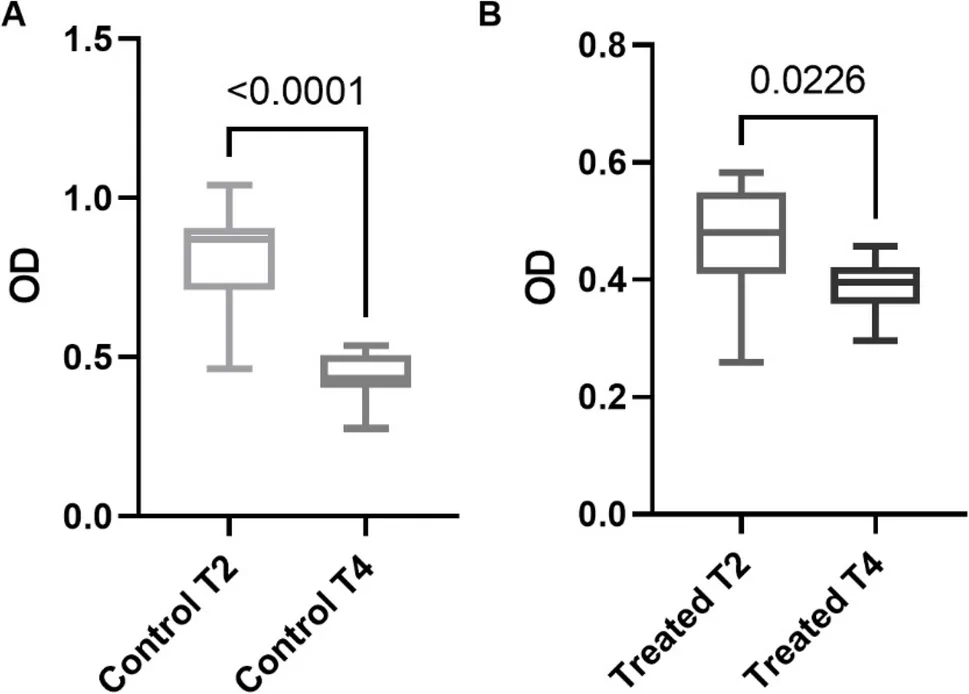
(**A**) IgG serum levels in control piglets for F4 between T2 and T4; (**B**) IgG serum levels in treated piglets for F4 between T2 and T4

**Fig. 4:**
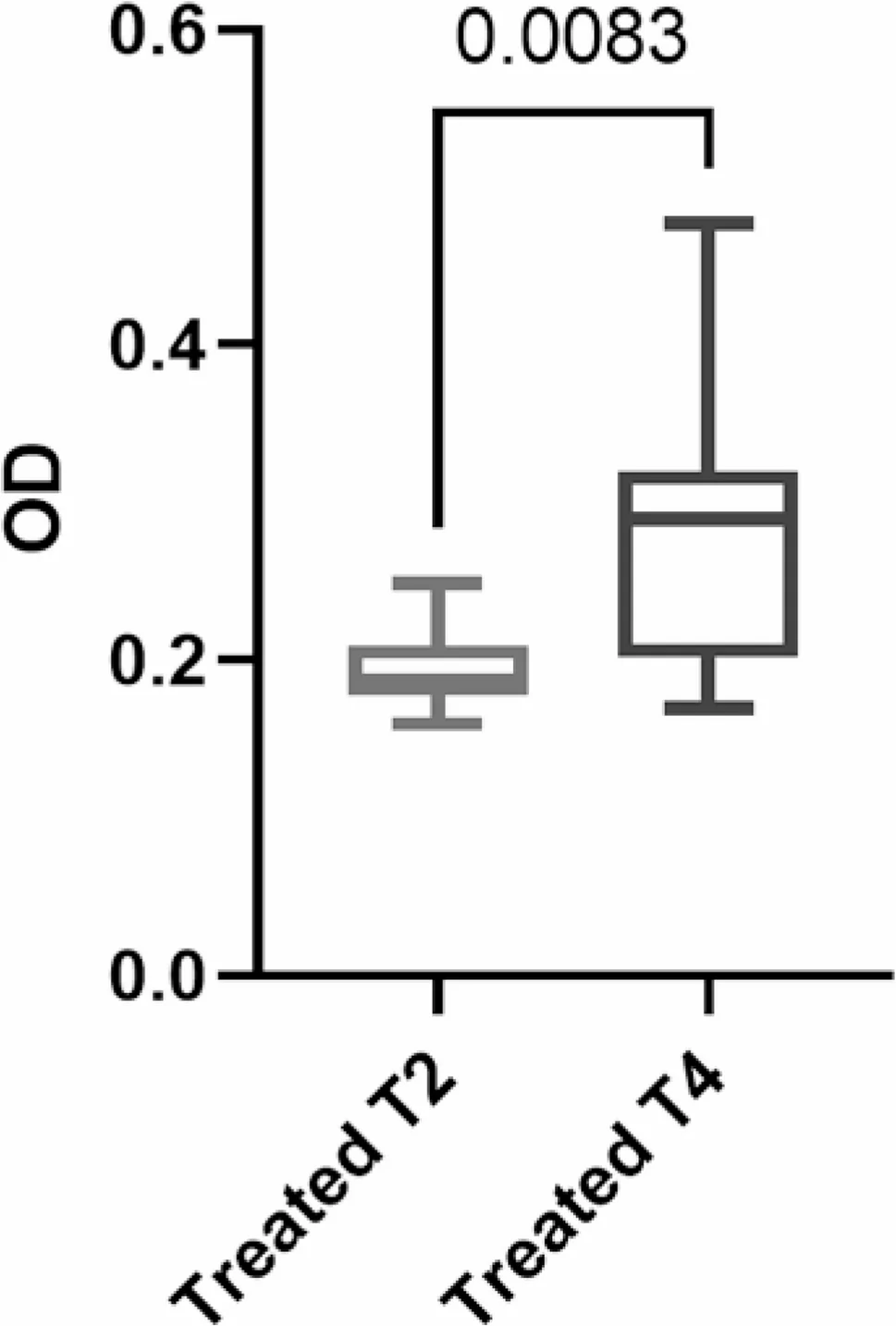
IgG serum levels in treated piglets for F18 between T2 and T4

Regarding anti-F4 IgG, significant differences between treated and control groups were detected at both T2 (*P* < 0.0001) and T4 (*P* = 0.0238), group C showing higher antibody levels than group T (Fig. 2A, B). Interestingly, the decrease of the IgG serum titers between T2 and T4 was less pronounced in treated, compared with control piglets (*P* < 0.0001 for group C and *P* = 0.0226 for group T) (Fig. 3A, B). Vice versa, the F18-specific IgG levels increased between T2 and T4 (*P* = 0.0083) (Fig. 4). However, no statistically significant differences were found between control and treated groups at T4 for F4, nor between control and treated groups at both T2 and T4 for F18. For completeness, the anti-F18 IgG data for the control group are presented in Supplementary Figure S3.

#### Salivary antibody response to the immunization

**Fig. 5:**
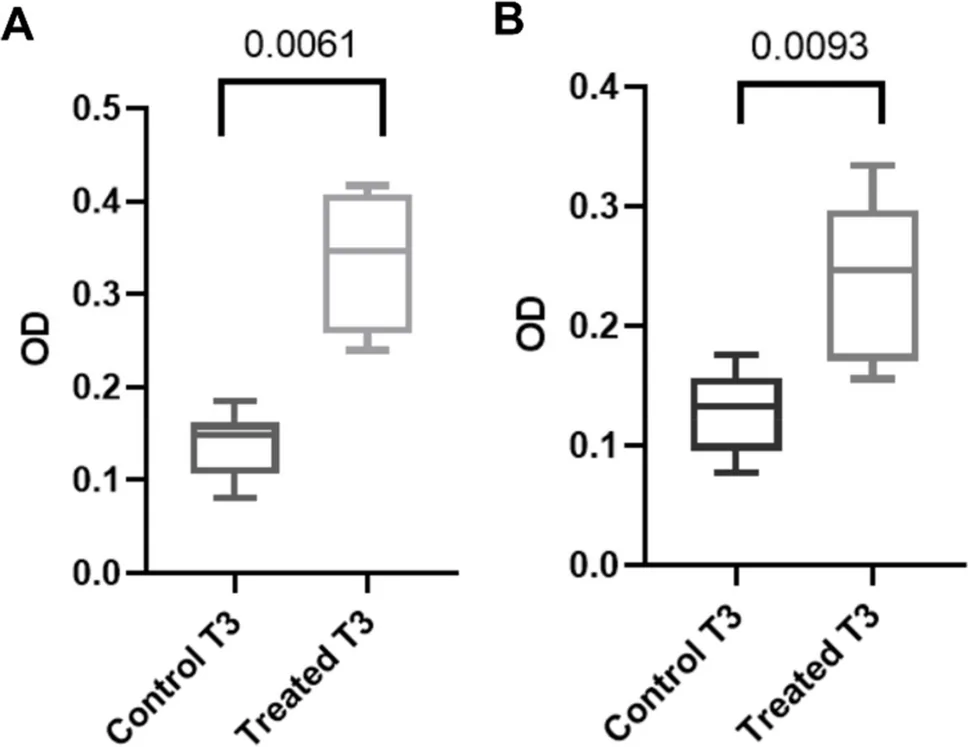
(**A**) IgA individual saliva levels in treated and control piglets for F4; (**B**) IgA individual saliva levels in treated and control piglets for F18

ELISA analysis revealed statistically significant differences in salivary IgA levels between T and C piglets at T3, seven days after the end of the administration of the immunizing product. Specifically, at T3 treated piglets displayed higher IgA levels against F4 (*P* = 0.0061) and F18 (*P* = 0.0093) compared to control piglets (Fig. 5A, B). Interestingly, salivary IgA Ab to fimbriae in the treated group peaked at T3 (34 days post-administration start) but declined rapidly by T4. No statistically significant differences in IgG levels against F4 were observed between the two groups at T3 and T4 (data not shown).

**Fig. 6:**
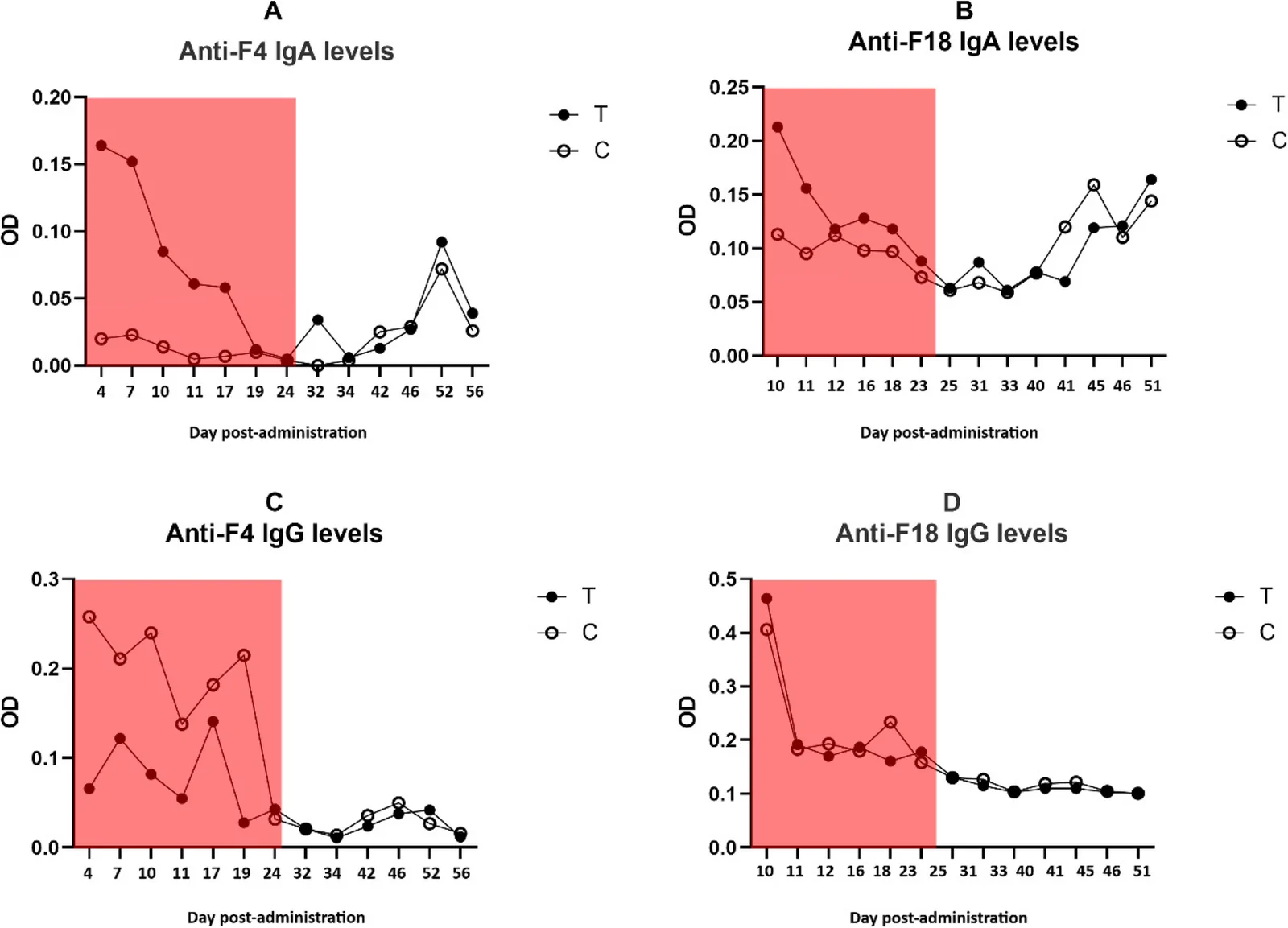
The optical density (OD) results from ELISA tests carried out on pooled saliva samples from litters of treated (T) and control (C) piglets to detect (**A**) anti-F4 IgA, (**B**) anti-F18 IgA, (**C**) anti-F4 IgG, (**D**) anti-F18 IgG levels during and following oral administration of the inactivated immunogenic product

Analyses on pooled saliva samples corroborated the trends observed in individual samples. Anti-F4 IgA levels (pooled samples) were initially higher in treated piglets during the first two weeks post-administration (4–17 dpa) but declined during the subsequent weeks (Fig. 6A). A second IgA peak was observed at 53 dpa in both groups. Anti-F18 IgA levels (pooled samples) followed a similar pattern of response, with a notable early peak in treated piglets. However, levels decreased around the middle of the trial, followed by a resurgence towards the end of the study, but in both groups (Fig. 6B). In contrast, anti-F4 IgG levels (pooled samples) were higher in control piglets during early sampling but declined significantly by 15.5-fold toward 34 dpa (Fig. 6C). Anti-F18 IgG levels (pooled samples) showed a parallel decrease in both groups over time, with no significant immunization effect (Fig. 6D).

Full dot, treated group; empty dot, control group. The light red box highlights the period of administration of the product.

#### Fecal IgA and IgG

**Fig. 7:**
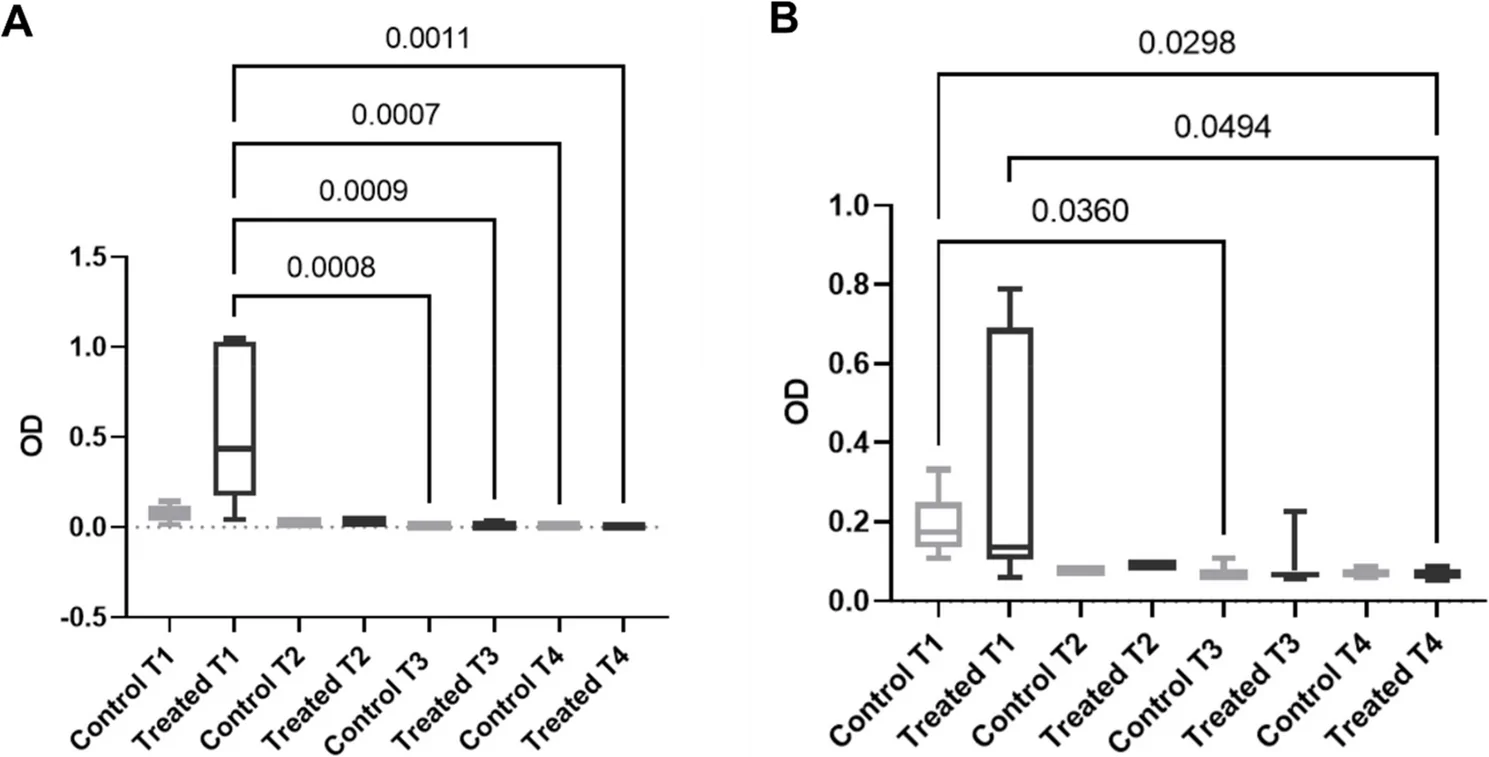
(**A**) IgA levels in feces of vaccinated and unvaccinated piglets for F4; (**B**) Fecal IgA levels in vaccinated and unvaccinated piglets for F18

In the case of fecal samples, slightly positive results were detected only at the first time point in terms of anti-F4 IgA, while at all other time points, the presence of Ag-specific IgA in feces was practically negligible. At the first time point, although data is not statistically significant, the treated group consistently showed higher IgA levels compared to the control group for both F4 and F18 (Fig. 7A, B). In contrast, anti-F4 and F18 IgG antibodies were undetectable in all fecal samples, as all ELISA results for IgG were negative.

### Optimization of ELISPOT for evaluation of IgA-secreting cells

**Fig. 8:**
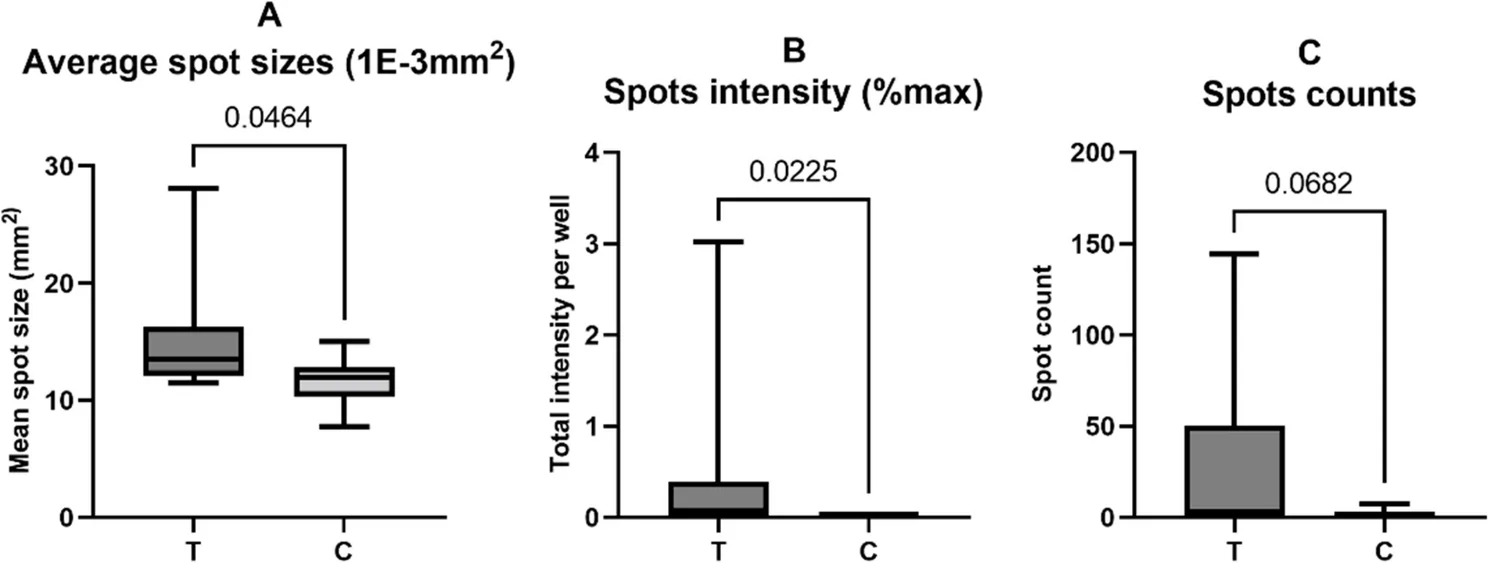
Boxplots of (**A**) average spot sizes generated by control and treated animals. In this calculation, the average values for each well are used without subtracting the unstimulated control values; (**B**) total intensity of all foreground objects minus background per well (% max) (Mann-Whitney, *P* < 0,05). After background noise subtraction, the foreground objects are analysed to identify spots of specific morphology, separating confluent and overlapping spots; (**C**) spot counts (Mann-Whitney, *P* < 0.1). In ELISPOT assay, spots of various sizes and densities are observed, depending on the different quantities and kinetics of Ig produced by the individual B cells

To further investigate the immunogenic capability of our product we set up an ELISPOT assay. Our primary focus was the quantification of IgA-secreting B cells isolated from the mesenteric lymph nodes of both treated and control piglets. First, we optimized the isolation of the mononuclear cells from the lymph nodes by the definition of the most adequate ratio between number of cells and a 3-mL Ficoll-Paque cushion volume. The most efficient number of cells was 71 × 10^6^, which allowed us to recover on average 5 × 10^6^ live mononuclear cells. Other parameters were optimized following the manufacturer’s protocol. We concluded that 5 days of pre-stimulation represented the optimal time for stimulation with both the polyclonal stimulus (Poly-S) and the specific stimulus (fimbriae). The optimal concentration of the Poly-S was 1:1000 and 2 µg/mL was adopted for purified F4 and F18 antigens. The most suitable number of cells to be plated was 4 × 10^5^ cells/well in order to obtain a readable number of spots. Table S4 summarizes all the tested conditions and parameters and highlights the optimal ones. Once the ELISPOT protocol had been set up, we proceeded with the analysis of all the samples. We analyzed three crucial parameters for each sample: the average spot size, the total intensity/well and the number of spots/well. We observed a statistically significant increase of the average spot size induced by the Poly-S in group T compared to C (*P* = 0.046; Fig. 8A). To gauge the amount of IgA secreted by cells accurately, the total intensity parameter, representing the difference in the foreground minus background for each well (% max), was evaluated; this revealed a statistically significant difference between group T and C (*P* < 0.05) (Fig. 8B). This parameter is pivotal to distinguishing between background signals and true spots. Additionally, a tendency was observed for treated animals to produce more spots per well compared to control animals, although the difference was not statistically significant (Fig. 8C).

Finally, there was a significant difference in the number of piglets that responded to the specific stimulus F4: in particular 5 treated piglets compared to 1 control (Chi square analysis, *P* < 0.05). Moreover, the intensity of spots was higher in the cells from the lymph nodes of treated piglets stimulated with F4 compared to controls (data not shown).

## Discussion

Weaning is a critical and stressful transition period for piglets, during which dietary changes, social reorganization and environmental stressors compromise immune defenses [3]. This heightened vulnerability facilitates *E. coli* colonization and increases the risk of gastrointestinal infections [4, 40], which makes a case for effective strategies to support immune resilience and disease prevention. In piglets, several *E. coli* strains cause diarrhea after weaning, leading to the reduction of production performances and an increased use of drugs [41]. This highlights the need for effective immunization protocols aimed at disease prevention and reduction of drug usage on farms. Current vaccination approaches primarily target gestating sows [42] toward the generation of high-titered specific antibody in colostrum and milk in the neonatal period; yet such antibody is unlikely to provide a satisfactory level of protection during the critical post-weaning period. In this respect, a major breakthrough was the development of the live, attenuated, Coliprotec F4 vaccine for piglets at weaning, which has proved effective in a controlled field study [43]. Yet, such a vaccine is not compatible with possible antibiotic treatments performed in “problem” farms, as a result of a strong environmental infectious pressure. Therefore, we reasoned that an oral, inactivated vaccine around weaning could be better suited to such farms, in agreement with the results of a previous study on deep creep feed containing inactivated *E. coli* cells [31]. The advantage of such an approach, in addition to being compatible with concomitant antibiotic treatments, is that the production of the oral inactivated product is cheap, and it can be administered easily with the feed or drinking water. Administration over several days can undoubtedly raise concern by farmers. In reality the proposed immunization protocol is not more demanding, in terms of manpower and infrastructure, than common mass administrations of drugs or feed supplements; moreover, the availability of water-powered proportional dosing pumps could enable vets to effectively perform mass administration of the inactivated product with minimal labor requirements.

Owing to the above, the present study evaluated the immunogenicity of an experimental, heat-inactivated, oral *E. coli* product to protect pigs against F4/F18 ETEC-induced post-weaning diarrhoea. Low-dose human IFN-α was selected as product adjuvant because of its proven effects in the prophylaxis of PWD [44], also recognized by EFSA in an official technical document [45]. After the integrity of fimbriae in our immunizing product had been confirmed, piglets were administered the product over 26 days, its immunogenicity being assessed later on. The administration of the product for almost a month could represent a disadvantage and be difficult to accept by the farmers. Yet, our study highlighted that already after 10 days of treatment the oral inactivated product could lead to a substantial mucosal IgA response to both F4 and F18 fimbriae.

Both sows under study had elevated levels of fimbriae-specific IgA and IgG in their colostrum, suggesting a previous natural environmental exposure to *E. coli*, despite a negative fecal PCR result. Indeed, PCR analysis on the sows’ feces aimed to exclude ETEC shedding by the animals enrolled in our study, but not a previous natural exposure to the pathogen. In newborn piglets, detectable levels of anti-F4 and anti-F18 IgG in serum, as well as IgA in mucosal secretions, are consistent with maternal transfer of immunity. This aligns with the established understanding that piglets rely on maternal colostrum and milk for passive immunity due to the epitheliochorial placenta in sows, which precludes transplacental transfer of immunoglobulins [46]. Interestingly, treated piglets showed a transient serum IgA response to F4 at T2, but exhibited lower serum IgG levels compared to control piglets. The presence of fimbriae-specific IgA and IgG in sow colostrum was unexpected, given the absence of clinical ETEC outbreaks and routine PCR-based screening of sows’ fecal samples for F4 and F18 ETEC strains until farrowing. This colostrum specific IgA and IgG could represent a maternal response to the ubiquitous presence of *E. coli* in the environment that may contribute to low-level antigen exposure and the development of pre-existing immunity in sows. Furthermore, the reduced systemic IgG response observed in treated animals might reflect an immunomodulatory effect of IFN-α, which has been shown to favor IgA class switching at mucosal sites, in agreement with a published in vitro model of human IFN-α in swine tonsil cells [47]. Initial IgA peaks observed in saliva samples likely reflect the presence of maternally derived antibodies (MDA) transferred via colostrum and subsequently transported into the gingival crevicular fluid [48]. The subsequent fluctuations of IgA levels over time are consistent with the activation of mucosal immunity in piglets following the treatment protocol. This pattern aligns with the significant IgA responses of treated piglets to F4 and F18 at T3, a few days after the immunizing treatment was over; the non-significant results at T4 indicates the rapid decrease of the Ab titers, after the removal of the immunizing product from the piglets’ diet. Instead, no statistically significant differences in anti-F4 IgG levels in saliva samples were detected between the groups at T3 and T4. Moreover, the ELISA results on pooled saliva samples confirmed the above findings, showing a clear IgA response to F4 and, to a lesser extent, to F18 in the first two weeks of treatment. The findings at later times on pooled saliva samples indicate instead the circulation of the pathogen once piglets were relocated from a monitored indoor space to an outdoor environment. These results must be considered in the light of the widespread presence of *E. coli* in the environment, including ETEC agents expressing F4 and F18 fimbriae [1, 14, 22, 41, 49–51].

The ELISPOT assay is considered the most suitable method for the assessment of cellular immune responses in the intestinal mucosa [52]. The stimulation of cells with F4 and F18 did not yield IgA spots in the majority of the animals, which is in agreement with the time-course of the mucosal IgA Ab response, already negligible at the time of lymph node collection. Yet, the ELISPOT assay data indicate that the product somewhat stimulated the piglets’ IgA production: the observed increase in spot size, spot intensity and number of spots in response to Poly-S in the treated group points at a measurable activation of total IgA-secreting B cells. This potentially highlights the efficacy of IFN-α as adjuvant of mucosal immune responses, in agreement with the aforementioned study [47]. A clearer ELISPOT response was reported in the study of Nguyen et al. [24], where they demonstrated that oral immunization in pigs with fimbriae, in the presence of MDA, can trigger an active immune response. However, they employed an ELISPOT assay on enriched IgA + B cells and found a notable increase in F4-specific IgA+ antibody-secreting cells (ASCs) following the second oral immunization with the specific antigen.

As for our study, the analysis of the Ab-positive samples seems to confirm that F4 is more immunogenic than F18, in agreement with other research findings [53].

The absence of fecal IgG Ab to fimbriae was expected, since such antibody is not likely to resist proteolytic degradation in the gut; the low-titered IgA+ fecal samples at T1 (treated group) may simply indicate that treated pigs possibly received a greater amount of colostrum and milk IgA antibody to fimbriae, compared with controls.

Some limitations were encountered in the study. To begin with, a 3rd experimental group.

given inactivated ETEC strains expressing F4 and F18 fimbriae without IFN-α would have.

been conducive to a correct assessment of the adjuvant activity of this cytokine towards further improvements of vaccine formulation. This will be undoubtedly a priority in further studies. Yet, our experimental findings indicate that the 2-group layout of our study did not bear on the evaluation of vaccine efficacy as a whole.

Secondly, maternal blood was not collected for antibody measurements, which prevented the determination of exact maternal antibody titers. Additionally, serum collection from piglets at T1 was not performed, as the study’s initial focus was on mucosal immunity. Also, pooled saliva collection started 4 days after the beginning of the treatment, since pre-treatment collection was technically difficult; we maintain however that T0 and T1 individual saliva samples can serve as a correct base line. Instead, blood sampling was planned for later time points (e.g., pre-weaning at T2 and at T4) to examine systemic immunity in relation to mucosal antibody levels. At T1, oral fluid and fecal samples were collected, with saliva serving as a key immunological matrix. In view of future efficacy trials, a genotyping test of pigs for F4 and F18 receptor polymorphisms should be performed before randomizing animals into experimental groups, and genetically susceptible pigs should be selected for challenge studies. The absence of this step in our study may have contributed to the observed variability. Having in mind the above caveats, our study has nevertheless indicated a practical and affordable immunizing procedure against ETEC strains, sustaining post-weaning diarrhea in pig farms with high circulation of ETEC. In this respect, the duration of the Ab response to our product until 50 days of age (T3 samples) and a possible delay of weaning until 32 days of age could represent a valuable approach to the prevention of PWD, usually peaking in piglets around fifteen days after weaning [5]. Our study also showed technical strength: the ELISA protocol proved to be a sensitive and reliable tool for quantifying antibody responses, with high reproducibility and minimal variability. Strong correlation coefficients (R² > 0.98) for positive controls and low coefficients of variation (< 10%) among replicates underscore the robustness of the assay. These technical strengths enhance the reliability of our findings and confirm the assay’s suitability for evaluating mucosal and systemic immune responses in vaccine studies.

## Conclusions

The development of new oral immunizing products is essential to improve intestinal health and reduce the incidence of post-weaning diarrhea (PWD) caused by ETEC in piglets. This preliminary study focused on the formulation and evaluation of a heat-inactivated oral immunizing product expressing F4 and F18 fimbriae. Immunoassay techniques, particularly ELISA and ELISPOT, provided critical insights into the immunogenicity of the product by detecting antigen-specific IgA and IgG responses in various biological matrices. With respect to Porter’s experimental model [31], our approach utilised a formulation system capable of preserving the antigenic integrity of fimbrial structures, thereby focusing the immune response to the fimbriae rather than on somatic components such as the O antigen. Despite this difference, significant similarities emerged. In both cases, no significant serum responses were obtained, indicating a limited systemic response. Also, mucosal immune responses declined rapidly after antigen withdrawal, suggesting a lack of durable immunological memory at mucosal sites.

These results underline a key challenge in oral immunization: the induction of a long-lasting mucosal immunity in the absence of continuous antigen exposure. Future work will require validation of this immunization approach under controlled challenge conditions, that closely mimics field exposure conditions. In conclusion, advancing our knowledge in this area will support the development of safe, effective and user-friendly oral product formulations for the sustainable control of PWD in pig production systems.

## Supplementary Information

Supplementary Material 1.

Supplementary Material 2.

Supplementary Material 3.

Supplementary Material 4.

## Data Availability

The data used and/or analyzed during the current study are available from the corresponding author on reasonable request.
