# Supplementary material for: Development of an inactivated, oral immunogenic product against post-weaning colibacillosis caused by enterotoxigenic Escherichia coli in piglets
Source: BMC Vet Res. 2026 Sep 2;22:528. doi: 10.1186/s12917-026-05846-5 (PMC13560234; doi:10.1186/s12917-026-05846-5)
Supplement: Supplementary file 1 [file 12917_2026_5846_MOESM1_ESM.docx]

**Supplementary Table 1** Mean optical absorbance (OD) values measured by indirect ELISA for F4 and F18 fimbriae on inactivated bacterial suspension samples. For F4, the heat-inactivated bacterial preparation yielded net OD values ranging from 0.261 to 0.298 at lower concentrations (1×–10×), markedly exceeding both the negative control and the positive control. The indirect ELISA employed (Verdonck et al., 2004), based on antigen adsorption and mAb (IMM01) binding to bioactive F4 fimbriae (FaeG subunit), ensures selective detection of structurally intact adhesins. The marked signal reduction at 20× is therefore likely attributable to antigen overload and saturation effects at the solid phase, leading to suboptimal antibody binding. In contrast, the F18 preparation exhibited net OD values between 0.047 and 0.067, only marginally above the negative control and markedly below the positive control. The absence of a validated monoclonal antibody capable of discriminating between bioactive and non-bioactive forms of the F18 fimbrial subunit (FedF) limits the structural assessment of F18 fimbriae and may partially account for the reduced signal intensity.

| Samples | Fimbriae |  | O.D. |
| --- | --- | --- | --- |
| Heat-Inactivated bacterial suspension | F4 | 1X | 0,261 |
|  |  | 5X | 0,298 |
|  |  | 10X | 0,290 |
|  |  | 20X | 0,079 |
|  | F18 | 1X | 0,067 |
|  |  | 5X | 0,059 |
|  |  | 10X | 0,052 |
|  |  | 20X | 0,047 |
| Positive Controls | F4 |  | 0,106 |
|  | F18 |  | 0,603 |
| Negative controls | F4 |  | 0 |
|  | F18 |  | 0 |
