# Supplementary material for: Development of an inactivated, oral immunogenic product against post-weaning colibacillosis caused by enterotoxigenic Escherichia coli in piglets
Source: BMC Vet Res. 2026 Sep 2;22:528. doi: 10.1186/s12917-026-05846-5 (PMC13560234; doi:10.1186/s12917-026-05846-5)
Supplement: Supplementary file 2 [file 12917_2026_5846_MOESM2_ESM.docx]

**Supplementary Table 2** The mean Optical Density values of the IgA and IgG in sow pooled colostrum by ELISA assay. “T” means Treated group; “C” means control group.

| Antigen | Colostrum sample | IgA (O.D) | IgG (O.D) |
| --- | --- | --- | --- |
| F4 | Sow (T) | 2.015 | 1.304 |
|  | Sow (C) | 0.771 | 1.692 |
| F18 | Sow (T) | 1.676 | 0.449 |
|  | Sow (C) | 1.591 | 0.397 |

During the set up of the ELISA protocol we had the limitation of the lack of a positive serum sample. This problem was partly linked to the control of the African Swine Fever virus epidemic in Piemonte region. Following preliminary tests on colostrum samples, we consistently observed high IgG and IgA values in colostrum sample from the mother of the treated piglets. Since we did not have access to a conventional positive control for comparison, the use of this colostrum provided a benchmark for relative comparison in each plate.

A summary table of the positive control results was reported:

| O.D F4 | | O.D F18 | |
| --- | --- | --- | --- |
| IgG | **IgA** | **IgG** | **IgG** |
| 1.30 | 2.02 | 0.66 | 1.29 |
| 1.18 | 1.86 | 0.51 | 0.97 |
| 0.94 | 1.06 | 0.45 | 1.68 |
