# Supplementary material for: Development of an inactivated, oral immunogenic product against post-weaning colibacillosis caused by enterotoxigenic Escherichia coli in piglets
Source: BMC Vet Res. 2026 Sep 2;22:528. doi: 10.1186/s12917-026-05846-5 (PMC13560234; doi:10.1186/s12917-026-05846-5)
Supplement: Supplementary file 3 [file 12917_2026_5846_MOESM3_ESM.docx]

**Supplementary Table 4** Results table for the optimization phase of ELISPOT assay. It was determined that five days of pre-stimulation with a control (unstimulated), polyclonal stimulus at a dilution of 1:1000, or F4 or F18 at a concentration of 2 μg/mL and 4x10^5^ cells/well were the optimal conditions. The selection of these parameters enabled the generation of a sufficient number of IgA-secreting cells for analysis and the identification of individual responsive cells, facilitating the differentiation from artefacts present within the well.

| **Number of days of pre-stimulation** | 4 days | **5 days** | 10 days |  |
| --- | --- | --- | --- | --- |
| **Kind of stimuli** | **unstimulated** | **Polyclonal stimulus: Poly-S** | **F4** | **F18** |
| **Dose of stimuli** | 0,1 µg/mL | **2 µg/mL** |  |  |
| **The number of cells** | From 1,5x10^4^ cell/well | **4x10^5^**  **cells/well** | To 1x10^6^ cells/well |  |
