# Supplementary material for: Development of an inactivated, oral immunogenic product against post-weaning colibacillosis caused by enterotoxigenic Escherichia coli in piglets
Source: BMC Vet Res. 2026 Sep 2;22:528. doi: 10.1186/s12917-026-05846-5 (PMC13560234; doi:10.1186/s12917-026-05846-5)
Supplement: Supplementary file 4 [file 12917_2026_5846_MOESM4_ESM.docx]

**Supplementary Figure 3.** IgG serum levels in control piglets for F18.
